# Mothers' Mobility after Separation: Do Grandmothers Matter?

**DOI:** 10.1002/psp.2010

**Published:** 2016-01-19

**Authors:** Marjolijn Das, Helga de Valk, Eva‐Maria Merz

**Affiliations:** ^1^ Statistics NetherlandsThe HagueThe Netherlands; ^2^ Netherlands Interdisciplinary Demographic Institute/University of GroningenThe HagueThe Netherlands; ^3^ Vrije Universiteit BrusselBrusselsBelgium; ^4^ Sanquin Blood SupplyAmsterdamThe Netherlands; ^5^ Vrije Universiteit AmsterdamAmsterdamThe Netherlands

**Keywords:** divorce, mobility, intergenerational relations, coresidence, the Netherlands

## Abstract

Starting from a life course perspective, this study aims to gain more insight into mobility patterns of recently separated mothers, focusing especially on moves to the location of their own mother: the maternal grandmother. Separated mothers, having linked lives with their own mothers, may benefit from their practical and emotional support. Additionally, the grandparents' home can be a (temporary) place to stay shortly after divorce. Data come from the System of social statistical datasets (Statistics Netherlands). This unique dataset combines longitudinal data from a vast number of administrative registers. It covers the complete Dutch population, making it exceptionally well suited for life course and mobility research. We studied mothers with minor children between 1/1/2008 and 31/12/2010. Our study included 579,500 mothers, of whom about 8,800 (1.5%) experienced a separation in 2008. Separated mothers moved to the grandmother's municipality more often than non‐separated mothers, which might be partially motivated by the need for childcare. They also coresided with the grandmother more than non‐separated movers, mostly because of a vulnerable socio‐economic position. Although often temporary, coresidence appears to have a prolonged impact on the mothers' location choice; mothers frequently stayed in the grandmother's municipality after moving out. Finally, our results indicated that some mothers seemed to use the parental home as a stepping stone to cohabit with a new partner. © 2016 The Authors. Population, Space and Place published by John Wiley & Sons Ltd.

## Introduction

Based on a life course perspective and linked family lives (Elder, 1994), women are important providers of intergenerational support and care (Pebley & Rudkin, 1999; Vandell *et al*., 2003; Knijn & Liefbroer, 2006; Fergusson *et al*., 2008; Hank & Buber, 2009). Especially grandmothers are key in giving practical support to their children and grandchildren, in accordance with the notion of ‘women as kinkeepers’ (Rossi & Rossi, 1990; Knijn & Liefbroer, 2006; Hank & Buber, 2009). Intergenerational ties and linked lives play an important role in mobility choices in general (cf. Coulter & Scott 2015; Coulter *et al*., 2015; Findlay *et al*., 2015). And particularly, different studies – among others in the Netherlands – have shown that parents' place of residence is relevant for spatial mobility decisions of adult children after divorce (Michielin *et al*., 2008; Smits, 2010; Smits *et al*., 2010; Mulder & Wagner, 2012; Mulder *et al*., 2012).

The break‐up of a romantic relationship is inextricably linked to mobility (Fischer & Malmberg, 2001; Feijten & van Ham, 2007). One of the partners will usually move to another place. In this study, we focus on mobility choices of recently separated mothers and address two main questions. First, we examine the role of linked lives (Elder, 1994) between separated women and their own mothers for mobility choices. Second, we aim to explain how support needs, indicated by life course characteristics of the separated mother (socio‐economic position, family size, and age of children), shape these mobility patterns. This study adds to existing knowledge by zooming in on *separated* mothers and their linked lives with their children and mother, as well as a new partner. Additionally, we examine coresidence (moving in with the grandmother) and moves close to the grandmother simultaneously. In contrast to most of the existing literature, we cover separations of both married and cohabiting mothers, doing more justice to the situation in most north‐western European countries where more and more children are born to unmarried couples.

The data for our study come from a unique combination of longitudinal registers in the Netherlands, the System of social statistical datasets (SSD; Statistics Netherlands). These individual‐level data, containing life course and geographical information, cover the full population of the Netherlands, allowing us to study all separated mothers (from either a married or unmarried union) with minor children.

## A Life Course Perspective on Intergenerational Relations after Separation

Intergenerational relations refer to solidarity between generations (Hammarström, 2005), most often between parents and their children (Silverstein *et al*., 1998) over the course of life. In his theoretical elaboration on life course studies, Elder (1994) identified that individual lives are linked to that of others. For example, relationships with primary caregivers, often the parents, guide individuals in their adult relationships. A harmonious parental home may pave the way for a positive view of family life, for having own children, and for reciprocal support exchange between partners and younger and older generations (Merz *et al*., 2007). Separation may have short‐term as well as long‐term negative consequences for ex‐partners (Amato & Keith, 1991; Joung *et al*., 1997; Murphy *et al*., 1997; Metsä‐Simola & Martikainen, 2013) and their children (Amato, 2000; Fischer, 2004; Feeney & Monin, 2008; Van Gaalen & Stoeldraijer, 2012).

Intergenerational ties within linked family lives are crucial. Grandparents continue to provide their children with emotional security and help them with caring for grandchildren (Pebley & Rudkin, 1999; Vandell *et al*., 2003; Knijn & Liefbroer, 2006; Fergusson *et al*., 2008; Hank & Buber, 2009). After separation, intergenerational ties between adult children and their parents might become more important because of the increased practical and emotional support needs of single mothers associated with the loss of the partner bond. Indeed, grandparents are more likely to help when their adult child is a single parent (Hank & Buber, 2009; Jappens & Van Bavel, 2012). Support by grandparents may be beneficial not only for the parent but also for the grandchildren. Grandparents have been shown to compensate for the lack of parental resources in children's school success (Jaeger, 2012). In line with theories on women as the main kinkeepers (Kalmijn, 2007), studies find that grandmothers offer more support than grandfathers (Knijn & Liefbroer, 2006; Hank & Buber, 2009) and maternal grandmothers are more active caregivers than paternal grandmothers (Hank & Buber, 2009).

Although the role of life course events for housing careers has been documented since the work by Rossi (1955), so far, little is known on the role of the grandmother's location for mobility decisions of recently separated mothers. Because proximity of family members facilitates contact and support (Joseph & Hallman, 1998; Smith, 1998; Hank, 2007; Mulder & Van der Meer, 2009), it may be advantageous for mothers to move near her parents after separation. Previous studies in the Netherlands found that divorced adult children more often move in the direction of their parents than married children, especially just after the divorce (Michielin *et al*., 2008; Smits, 2010). Similarly, parents of young children more often move close to their own parents rather than settling elsewhere (Smits, 2010). In general, moves closer to the family seem to be motivated by emotional and practical support needs. Therefore, we expect that *overall, separated mothers move more often than non‐separated mothers, and that separated mothers are relatively likely to move to the municipality of the grandmother compared with non‐separated mothers who move* (H1).

Another way in which parents help their children across the life course is providing a home. Adult children sometimes (temporarily) move back in with parents during times of transition or difficulty (Da Vanzo & Goldscheider, 1990; Smits *et al*., 2010; Dykstra *et al*., 2013). After separation and difficulties to find independent housing, coresiding with the parents for a limited period of time may prove a practical solution and provide adult children with additional emotional support and security within their former family home. Hence, we hypothesise that *recently separated mothers are more likely to move to the grandmother's home compared with non‐separated mothers who move* (H2).

Intergenerational solidarity including parental and grandparental care is key in family dynamics. Young children need the most intensive daily care. Reconciling work and family becomes easier as children grow up. Child age and parity shape the mother's support needs: a larger family means a larger investment in daily care. We therefore hypothesise that s*eparated mothers with younger children, especially preschoolers, and those with more children are more likely to move to the municipality of the grandmother* (H3).

Generally, coresidence is more common among adult children without employment and economically more disadvantaged households (Grundy, 2000; Choi, 2003; Hank, 2007; Smits *et al*., 2010). This association may be even stronger in case of a separation, owing to associated income loss. We predict that *separated mothers with an unfavourable economic position, that is, low income and unemployment, will more often move in with the grandmother than those who are well off* (H4).

If the mother becomes involved in a new romantic relationship, her life is linked not only to her own parents and children but also to the new partner, which will influence her decisions regarding mobility. Cohabitation with a new partner was found to mitigate negative effects of divorce for adults by offering support and reducing loneliness (Amato, 2000) and is associated with better housing conditions (Feijten & Van Ham, 2010). Having a new partner may also mean sharing employment, housework, and childcare. We expect that mothers who cohabit with a new partner after separation need less intergenerational support. In other words, *separated mothers who have started a cohabitation with a new partner will be less likely to move to the municipality of the maternal grandmother* (H5).

### The Dutch Context: Family Relations, Care for Children, and Union Dissolution

Distances between family members are relatively limited in the Netherlands, a small and densely populated country. The average distance between adult children and their parents is 30 km (Mulder & Kalmijn, 2006), and about half of parents live within 5 km of their children (Van der Pers & Mulder, 2013). The highest levels of support are found when parents and children live close and decrease with increasing distance (Knijn & Liefbroer, 2006; Mulder & Van der Meer, 2009).

In 2010, Dutch municipalities averaged less than 80 km^2^ (Statistics Netherlands), meaning that the distance between mothers and grandmothers living in the same municipality is hardly more than 10 km and generally less. Dutch towns have an excellent infrastructure for bicycles and pedestrians, and larger towns also have good public transport facilities, making the exchange of practical support within municipalities quite easy.

Intergenerational contact and support are relatively high in the Netherlands compared with those in other European countries (Hank & Buber, 2009). About 75% of Dutch parents have weekly contact with at least one of their adult non‐coresident children (Kalmijn & Dykstra, 2006), and 50% of parents provide some type of intergenerational support (Knijn & Liefbroer, 2006). Contact frequencies even increase when there are grandchildren (Kalmijn & Dykstra, 2006). The most common types of grandparent support include financial support, help with household chores and odd jobs, and childcare (Knijn & Liefbroer, 2006; Geurts *et al*., 2012).

Unmarried cohabitation and having children out of wedlock are quite common in the Netherlands. There are standard legal arrangements available for unmarried couples to arrange (financial) rights and obligations, legal paternity, and shared custody (Schrama, 2008; Poortman & Mills, 2012).

Although many childless couples have a relatively equal division of tasks (in terms of both paid and unpaid work), this changes drastically when children are born. In 2011, 45% of first‐time mothers either stopped working or substantially reduced their paid working hours (Cloïn & Bierings, 2012). Only 30% of households with primary school children use formal childcare, and mothers spend more time taking care of children than fathers do (Cloïn & Souren, 2011; Cloïn & Bierings, 2012). As a result, mothers of minor children are often economically dependent, making them more vulnerable in case of a separation. After divorce, the majority of children live with their mother. In 2008, 16% of the children had dual residency, living alternately with the mother and the father, and a small minority (less than 5%) lived with the father (Spruijt & Duindam, 2009).

## Data and Methods

### Dataset and Study Population

To study mobility patterns after separation, we use the SSD of Statistics Netherlands (Bakker *et al*., 2014). The SSD combines various administrative registers, among which are the population register and tax registers, covering the complete Dutch population. Because the SSD is longitudinal and contains information on location, distances, and mobility, it is exceptionally well suited for life course and mobility research.

Our study focuses on *de facto* separations of both married and cohabiting mothers between 1/1/2008 and 1/1/2009 and their moves until 31/12/2010. The latter year covers the most recent information available at the time of study. The majority of moves after a separation take place within the first couple of months. The frequency of moving then gradually declines, and we observed very low mobility of separated mothers in the second half of 2010. We take a larger time window to cover broader mobility patterns after separation, not just the first move. A quarter of all separated women moved at least twice within the 2–3 years after separation. Married and unmarried cohabitation and separation were defined using both partners' addresses. We do not measure *de jure* divorces as we include both married and unmarried cohabiting mothers, and also because the timing of *de jure* divorce is not suited to specifically capture the moves that initiated the actual separation, the splitting up of the household.^1^

Our starting point was mothers with minor children whose family was intact at 1/1/2008 and whose own mother was alive. Mothers already living in the municipality of the grandmother before separation – around half of all mothers – were excluded from the analyses. Additionally, we excluded a small number of mothers who had emigrated or died and/or whose partner had died or emigrated in 2008. Last, a small number (13%) of separated mothers whose children either were registered at the father's address or lived elsewhere (e.g. independently) were excluded. Hence, our sample includes both mothers who were the main caretakers and mothers who share childcare with the father but whose children were registered at her address. Our study population consisted of 579,500 non‐separated mothers^2^ and 8,800 separated mothers.

### Dependent and Independent Variables

Our dependent variable ‘mobility patterns’ is primarily based on mother's location at 31/12/2010, as we aim to also capture the more long‐term residential choices. Choices with respect to coresidence were the exception as they were usually temporary with a median duration of 8 months. Eighty‐five per cent of coresiding mothers moved at least twice, as opposed to only 20% of non‐coresiding separated mothers. In order to capture even short periods of coresidence, we constructed specific categories for those cases in which mothers moved to coresidence as a first or second move^3^ but did not live in coresidence at the end of the observation period.

We observed non‐separated mothers from 1/1/2008 to 31/12/2010 and separated mothers from the moment of separation (taking place in 2008) to 31/12/2010. The move indicating the separation itself was included as the most relevant one for studying temporary living arrangements such as coresidence.^4^

Hence, our dependent variable consisted of seven categories:

1. ‘moved to grandmother's municipality’: moved during observation period and lived in grandmother's municipality at 31/12/2010.^5^
2. ‘long‐term coresidence’: moved to coresidence during observation period and stayed there until 31/12/2010. The median length of long‐term coresidence in our study was 29 months. This figure reflects the fact that in most cases women moved to coresidence directly after separation.
3. ‘short coresidence, stayed in grandmother's municipality’: spent any time in coresidence during observation period and moved out again. Lived in the grandmother's municipality at 31/12/2010.
4. ‘short coresidence, left grandmother's municipality’: spent any time in coresidence and moved out again during observation period, leaving the grandmother's municipality.
5. ‘moved within municipality’: moved during observation period and, at 31/12/2010, lived in the same municipality as before separation (not the grandmother's municipality).
6. ‘moved between municipalities’: moved and lived in another municipality (not the grandmother's) at 31/12/2010.
7. ‘non‐movers’: did not move in observation period.

The distribution of the main independent variables, defined based on our theoretical assumptions, is shown in Table 1. The independent variables included the following: *children's age* (age of oldest minor child), with three categories [preschool (aged 0–4), primary school (aged 4–12), and adolescent (aged 12–18)]; *family size* (one child in the household, two children, and three or more children); yearly household *income* in deciles, including both the fathers' and mothers' income before separation; *employment* status of the mother [working in a paid job before the separation (yes/no)]; and presence of a *new partner* in the household (yes/no). All independent variables were measured before separation at 1/1/2008, except presence of a new partner, which was measured at 31/12/2010.

**Table 1:** Distribution of study variables (% per category, by union status).

	Definition/categories	Not separated (98.5%)	Separated (1.5%)
Children's age (age of oldest minor child)	Preschool (aged 0–4) (reference)	23	29
	Primary school (aged 4–12)	42	46
	Adolescent (aged 12–18)	35	26
Family size	One child in the household	27	37
	Two children (reference)	51	46
	Three or more	23	18
Household income	Yearly income of the household in deciles	Median €22,675	Median €20,204
Employment of mother	Not employed (reference)	30	33
	Employed	70	67
New partner (2010)	No new partner (ref)	Not applicable	77
	New partner	Not applicable	23
Controls			
Mother's age	Age under 25	1	6
	Age 25–29	7	11
	Age 30–34	18	19
	Age 35–39 (reference)	28	28
	Age 40–44	25	21
	Age over 44	21	13
Marital status	Married (reference)	83	60
	Never married	16	29
	Divorced or widowed	2	11
Relationship of grandparents	Grandparents together (reference)	63	56
	Grandparents separated	10	19
	Grandfather deceased, emigrated, or unknown	27	26
Initial distance of mother–grandmother (km)	Average Euclidean distance in kilometres between centroids of municipalities	42	40
Migrant status	Native Dutch (reference)	90	84
	Non‐Western, first or second generation	4	7
	Western, first or second generation, excluding native Dutch	6	8
Degree of urbanisation	Very strongly urban (reference)	12	15
	Strongly urban	27	29
	Moderately urban	22	22
	Rural	24	22
	Strongly rural	14	12
Total *N*		579,500	8,800

Note: Variables represent the situation at 1/1/2008 before the union dissolution, except the variable indicating whether a new partner was present (at 31/12/2010). Frequencies are rounded to 50s, for reasons of privacy and data protection.

Source: System of social statistical datasets, Statistics Netherlands (own calculations).

Next to income and employment, educational level is another important indicator of the socio‐economic position of the mother. However, in our data, educational level could only be determined for 46% of our study group. Given the large number of missing data and the fact that the subgroup for whom we know the educational level is highly selective – among other things, young mothers are strongly overrepresented – we have not included this indicator in the remainder of this study. Nevertheless, we performed several exploratory logistic regressions including educational level, both weighted and unweighted. These analyses suggest that our findings are quite robust: none of the coefficients changed substantially when including educational level in any of the exploratory models.

In addition to our key variables of interest, several control variables were included, based on theoretical considerations. *Mother's age* indicate that young mothers receive more support from grandparents than older mothers (e.g. Vandell *et al*., 2003; Knijn & Liefbroer, 2006; Fergusson *et al*., 2008) and younger persons are geographically more mobile (Feijten & Visser, 2005; Etzo, 2008). In our research population, mother's age was correlated to, among other things, her children's age, family size, income, and the probability that her own parents were alive. However, none of these correlations resulted in multicollinearity problems (as shown later).

*Marital status* indicates whether the mother was married or cohabiting. For cohabitation, we further distinguished between those who ‘never married’ and those who were previously ‘divorced or widowed’. This last category included previous divorces or widowhood as well as mothers who were already formally divorced but were still living together with that partner at 1/1/2008.

*Relationship status of the grandmother* indicates whether the grandmother lives with the maternal grandfather or not (further distinguished into separated and widowed grandmothers). Studies show that divorced parents support their adult children less and have less contact with them than married parents (Kalmijn & Dykstra, 2006; Knijn & Liefbroer 2006). Initial *distance* between the municipalities of the mother and the grandmother measured in kilometres is another control in the models. Distance between family members is important for the purpose of our study and has been shown to be related to socio‐economic status, household, and marital status (Mulder & Kalmijn, 2006; Michielin & Mulder, 2007). Last, all models were corrected for *migrant status* and *degree of urbanisation* of the women's location before separation (e.g. Smits, 2010; Smits *et al*., 2010). Correlations between each of the independent variables never exceeded 0.5, and no multicollinearity problems were found.

## Results

### Descriptive Findings: Moves of Non‐separated and Separated Mothers

We indeed find (H1) that separated mothers moved more often than non‐separated mothers (Table 2). Two‐thirds (68%) of separated mothers had moved at least once in the 2–3 years after separation, while only around one in 10 (11%) non‐separated mothers had moved in the same observation period.

**Table 2:** Mobility patterns of separated and non‐separated mothers (%).

	Non‐separated (%)	Separated (%)
Not moved	89	32
Total moved	11	68
Moved to grandmother's municipality	11	13
Long‐term coresidence	1	3
Short coresidence, stayed in grandmother's municipality	1	5
Short coresidence, left grandmother's municipality	1	4
Moved within municipality	56	50
Moved between municipalities	31	24
Total *N*	579,500	8,800

Source: System of social statistical datasets, Statistics Netherlands (own calculations).

When we compare the destinations of both separated and non‐separated mothers who move, we find that of all separated mothers who moved, around 13% moved to the grandmother's town (H1; Table 2 last column). Furthermore, another 5% had coresided with their mother for some time and were still living in the grandmother's town at the end of our observation. In addition, 3% coresided with the grandmother on a long‐term basis. Among non‐separated mothers, in total, 13% (sum of first rows in Table 2) of all movers were living in the grandmother's municipality at the end of 2010, in either coresidence or own housing.

A total of 12% of separated mothers who moved spent a period of time coresiding with the grandmother after the separation. In line with H2, we find this to be much less common among non‐separated mothers, of whom only 3% of the movers spent some time in coresidence between 2008 and 2010. Coresidence after separation was often followed by another move within the next years. After 8 months, 50% of coresiding mothers had moved out of the maternal home again, and at the end of 2010, 80% had moved out. This result is in line with the idea that coresidence is often a temporary solution to a housing problem. Nevertheless, this temporary solution may have a prolonged impact on location choice and settlement: of all separated mothers who coresided with the grandmother at any point after separation, almost half stayed in the grandmother's municipality after moving out of the grandmother's house.

Given the importance of distance in location choice and help from mothers, we explored the initial distance between municipalities of the mother and the grandmother before separation (results not shown). The findings show a clear relation: mothers who moved to the grandmother's municipality initially already lived closer to her than average. Also, mothers who moved to another municipality initially lived further away from the grandmother than mothers who moved within the municipality. These patterns were found to be similar for non‐separated and separated mothers.

Although mothers who already lived in the same municipality as the grandmother (*N* = 575,600) were not included in our analyses, we did carry out additional analyses on this group to have a better idea of who they are. These mothers had, on average, somewhat lower incomes (€20,400) than the mothers in our study population (€22,700), were slightly younger (median age 38 against 39), and more often of non‐Western origin, 7% against 4%. The occurrence of separation was very comparable: 1.6% separated in 2008 compared with 1.5% in our study population. Exploratory analyses showed that the mobility patterns of separated mothers who already lived near the grandmother fit with the general ideas behind this study. For instance, 9% of these separated mothers moved to coresidence, against 0.4% of non‐separated. Furthermore, separated mothers who already lived near the grandmother more often did not move at all in the observation period: 42%, as opposed to 32% in our study population of separated mothers who lived in a different municipality than the grandmother. This is in line with our general idea that grandmothers may fulfil an important support role in case of separation. For those already living in the maternal municipality, staying behind in the former joint home may be a good strategy when support is needed.

### Multivariate Results

In the next step, we carried out two multinomial logistic regressions, with mobility patterns as the dependent variable and including a dummy variable indicating separation as the main explanatory variable. These multivariate analyses confirm that the moving behaviour of separated mothers differed significantly from that of non‐separated. For testing whether separated mothers move more often than non‐separated mothers (H1), we take non‐movers as the reference category (Table 3). Indeed, we find that separated mothers moved more often. Additional analyses (not shown but available upon request) showed that moves to the grandmother's municipality and to her home (both long‐term and short‐term coresidence) were relatively more likely than moves to other municipalities, in line with H1 and H2.

**Table 3:** Multinomial logistic regression coefficients for mobility patterns of separated and non‐separated mothers.

	*B* coefficient of independent variable
	Separated (yes; reference = not separated)
Moved to grandmother's municipality	3.098***
Long‐term coresidence	3.703***
Short‐term coresidence, stayed in grandmother's municipality	4.785***
Short‐term coresidence, moved elsewhere	4.376***
Moved within municipality	2.774***
Moved between municipalities	2.739***
Not moved	Reference

Note: Nagelkerke pseudo‐*R* ^2^ = 0.124; *N* = 588,319. Control variables (age of oldest child, family size, income, employment, age, marital status, relationship of the grandparents, distance, migrant status, and urban/rural environment) are not shown. Full model available from the first author upon request.

Source: System of social statistical datasets, Statistics Netherlands (own calculations).

*** *p* < 0.001.

For the subpopulation of separated mothers, we performed a multinomial logistic regression on mobility patterns, testing the main effects of life course characteristics including children's age, family size (H3), income, employment (H4), and presence of a new partner in the household (H5). Compared with other between‐municipality movers, mothers of preschool children are indeed (H3) found to move to the grandmother's municipality more often than mothers of primary school children aged 4–12, but not more often than mothers of adolescents (Table 4). Short‐term and long‐term coresiding mothers did not differ significantly from other between‐municipality movers with respect to their children's age. In general, mobility patterns of mothers with preschool children differed from those of mothers with older children: mothers with preschool children moved more often between municipalities and less often within the municipality.

**Table 4:** Multinomial logistic regression coefficients of mobility by life course characteristics (separated mothers only).

	Moved to grandmother's municipality	Long‐term coresidence	Short coresidence, stayed in grandmother's municipality	Short coresidence, left grandmother's municipality	Moved within municipality
	*B* coefficient	*B* coefficient	*B* coefficient	*B* coefficient	*B* coefficient
Children's age					
Preschool (aged 0–4) (reference)					
Primary school (aged 4–12)	−0.259*	0.115	−0.139	0.028	0.584***
Adolescent (aged 12–18)	−0.319	−0.227	−0.739	−0.656	0.538***
Family size					
One child in the household	0.043	0.578*	0.265	0.174	−0.202*
Two children (reference)					
Three or more children	0.044	−0.538	−0.462	−0.408	−0.011
Household income					
10% lowest income	0.030	1.101	1.172**	0.348	−0.170
10–20%	0.288	1.403*	1.302**	0.750	−0.112
20–30%	−0.221	1.340*	1.124*	0.272	−0.196
30–40%	0.207	1.407*	1.599***	0.823*	0.139
40–50%	0.124	1.108	1.000*	0.174	0.004
50–60%	0.149	1.017	1.239**	0.741	0.116
60–70%	0.192	0.790	1.103*	0.326	0.061
70–80%	0.184	0.621	0.440	−0.387	0.047
80–90%	−0.029	0.659	0.883	0.290	0.294
10% highest income (reference)					
Employment					
Employed	0.018	−0.242	−0.082	0.103	0.269***
Not employed (reference)					
New partner					
No (reference)					
Yes	−0.873***	−2.813***	−1.038***	−0.110	−0.715***

Note: Reference category: moved between municipalities (not the grandmother's). Nagelkerke pseudo‐*R* ^2^ = 0.235, *N* = 5,964. Control variables (age, marital status, relationship of the grandparents, distance, migrant status, and urban/rural environment) are not shown. Full model available from the first author upon request.

Source: SSD, Statistics Netherlands (own calculations).

* *p* < 0.05,

** *p* < 0.01,

*** *p* < 0.001.

Our hypothesis that the probability of moving close to the grandmother is positively related to *family size* (H3) was not confirmed. Mothers who had only one child moved less often within their municipality and more often between municipalities. Movers to the grandmother's municipality and short‐term coresiders did not differ from other between‐municipality movers with respect to the number of children. Long‐term coresiders, however, more often had only one child than other between‐municipality movers.

In accordance with H4, long‐term coresiders and, especially, short‐term coresiders who stayed in the grandmother's municipality more often belonged to *low‐income groups* before the separation compared with other between‐municipality movers. Short‐term coresiders who left the grandmother's municipality on the other hand seemed to have a more advantaged socio‐economic position than other coresiders in terms of income. In contrast to expectations (H4), *employment* of the mother was not significantly associated with the probability of coresidence. Additional analyses on the role of educational level (results not shown but available upon request) support the idea that mothers who coreside seem to be a vulnerable group. On average, coresiders were lower educated than other separated mothers, especially long‐term coresiders. In contrast, separated mothers who moved to the grandmother's municipality were not an economically vulnerable group; their income and employment levels before separation were comparable with those of other between‐municipality movers. Movers within the municipality did not differ from between‐municipality movers with respect to pre‐separation household income but were more likely to be employed. This could be related to the fact that jobs may be locally based and represent location‐specific capital, which could inhibit moving over long distances.

Mothers who cohabited with a *new partner* at the end of 2010 more often moved between municipalities compared with moving within (Table 4). Confirming our hypothesis (H5), separated women with a new partner moved less often to the municipality of the grandmother and were less often coresiding for a long or short period. Surprisingly, mothers who coresided with the grandmother at some point and then moved out of that municipality again had a high probability of living with a new partner at the end of 2010: 42% of this group lived with a new partner as opposed to 23% of all separated mothers. This was comparable with the 38% between‐municipality movers who cohabited with a new partner at the end of 2010 but significantly higher than all other movers (results not shown). Further exploratory analyses showed that separated mothers who spent time in coresidence but moved out again were more likely to live in the grandmother's municipality at the end of 2010 than mothers who had not spent time in coresidence (*χ* ^2^ = 697.2; *df* = 2; *p* < 0.001).

Because the control variables in our models overall confirm findings from previous studies, we do not report on them here in detail. Full details are available from the first author upon request.

## Conclusion and Discussion

By taking a life course perspective, this study aimed to shed light on mobility patterns of recently separated mothers in the Netherlands, with a special focus on the links between lives of the mother, her children, her own mother, and her new partner. Our results add to existing knowledge in three ways. First, while previous studies analysed moving close to family in general, we focused specifically on *separated mothers*, an understudied group with regard to mobility close to family. Intergenerational support within ‘linked lives’ across the life course influences the location choice of separated mothers. Second, contrary to most other studies, we included divorced and unmarried cohabiting mothers in our study. Last, our study covered both moving close to and moving in with the grandmother. This approach gives more insight into interdependencies between these two types of moves and on causes and consequences of mobility.

Using unique data covering the entire population of the Netherlands, we examined whether separated mothers' mobility behaviour is influenced by linked lives. Indeed, we find that location of the grandmother, age of children, and a potential new partner were related to separated mothers' moving patterns.

### Does the Grandmother Matter?

We found that separated mothers were more likely to move to the grandmother's municipality than non‐separated mothers and in addition were also more likely to move in with the grandmothers. Previous studies already indicated that the location of the grandmother – or grandparents – matters for mobility decisions of families with children (Michielin *et al*., 2008; Smits, 2010), but our result showed that their location matters even more after separation.

Furthermore, mothers with preschool children were more likely to move to the grandmother's municipality than mothers of primary school children, reflecting their greater need for help with childcare. However, this effect was not linear as expected: mothers of adolescents were as likely to move near to the grandmother as mothers of preschoolers, possibly indicating a broader intergenerational support pattern. Mothers may be more concerned about adolescents' well‐being and adaptation after separation, given the sensitive phase their children are in. Furthermore, contrary to our expectations, family size did not matter: mothers with more children did not move to the grandmother's municipality more often than mothers with one or two children. The need for help with childcare may play a modest role in the decision to move close to the grandmother, but this is probably not the whole story. Other motivations may play a role as well. First, emotional support and security may be even more important than practical help with childcare. The need for emotional support does not diminish when children become older. Second, the motivation to move to the grandmother's town may be related to the location itself. In many cases, this town is the place where the mother spent her childhood. Part of the old social network, friends, and/or siblings may still reside there (Wall & Von Reichert, 2013). This might especially be true for young women, explaining the finding that younger mothers more often moved to the grandmother's municipality than older mothers.

### Moving in: Causes and Consequences of Coresidence

Providing temporary housing to adult children is one form of intergenerational support. Adult children may coreside with their parents because they have limited finances and are unable to quickly buy or rent a home of their own after separation. This is in line with other findings showing that economically disadvantaged adults more often live with their parents.

In contrast to the idea that coresiders are an economically vulnerable group, mothers who coresided were not more often unemployed than movers to other municipalities. But our results also show that employed women are generally less likely to move over longer distances (movers between municipalities are less often employed than movers within the municipality). Employment might tie women to a specific location and inhibit longer‐distance moves. To gain more insight into the role of employment for mobility decisions and coresidence, more in‐depth data are needed including job location and travel distances.

In addition, our results show that coresidence might not always be driven by mere economic necessity. The dynamics around coresidence are more complex, in both their causes and consequences. Three mobility patterns around coresidence can be distinguished. First, a minority of one in five mothers still lived in coresidence at the end of the observation period. These long‐term coresiding mothers generally had a weak socio‐economic position. Second, almost half of coresiders relocated within the grandmother's municipality after moving out. This suggests that the family has acquired location‐specific capital during the time spent in coresidence. Such local capital – for example, the social network of school – provides strong ties that bind the family to the place (Da Vanzo, 1981; Mulder & Wagner, 2012). Thus, an initially practical housing decision may determine the family's spatial location for a long time after. Finally, one‐third of all coresiders left the grandmother's municipality after moving out of the grandmother's home. This group is not economically vulnerable; their socio‐economic position is comparable with that of other separated mothers. For them, moving in with the grandmother does not seem to be motivated by a lack of financial options to buy or rent a home on short notice. A number of these coresiding mothers may have used the grandmother's home as a safe haven while making arrangements to live with a new partner. These relationships may already have existed at the time of separation. To gain more insight into these relationship transitions, survey data are needed that capture these dynamics in more detail.

### Limitations and Concluding Remarks

This study contributes to our understanding of mobility patterns after separation and provides evidence that location choice of mothers is influenced by intergenerational linked lives. Children and their social network tie the family to a location and may put a severe restraint on the mother's mobility. The father and his location may also influence mother's mobility after separation but was unfortunately beyond the scope of this study (Bakker & Mulder, 2013). Also beyond the scope of this study were the role and dynamics of the housing market, which may be an important factor in mobility choices of separated mothers across the country. For the future, population register data offer promising opportunities to elaborate further on partner dynamics and mobility after separation, especially in combination with survey data that provide information about day‐to‐day care and living arrangements.

Although register data do not suffer from selective nonresponse and problems of insufficient data, they have other limitations such as errors and sometimes suffer from administrative delay. In addition, some indicators would ideally be measured in more detail. This holds especially for distance between mothers and grandmothers, defined here as the distance between the centres of their municipalities. Moreover, because the data are collected for administrative purposes, they do not always represent reality as experienced by people themselves. Therefore, they are not suited to study some of the more complex aspects of social ties, such as the timing and evolvement of romantic relationships and parenting dynamics after divorce. Last, registers do not measure subjective information such as motivations, preferences, and attitudes. Examining whether moving behaviour is (partly) motivated by intangible benefits, such as emotional support, requires survey data or qualitative in‐depth interviews that give insight into the psychology of human choices.

Another interesting question, beyond the scope of this study, is whether children also benefit from moves near their grandmother. One negative consequence of parental divorce is that children often partially lose access to resources of the father (Fischer, 2004), and it would be interesting to investigate whether grandparental resources (care, involvement, and investments) could partly compensate for this loss.

Overall, we can conclude that grandmothers matter for the spatial decisions of mothers after separation. These are times of rising divorce rates, and there is a growing need for informal care in many countries where support from the state is either limited or dwindling because of government budget cuts. The importance of intergenerational support by family is, therefore, ever increasing. Studying how intergenerational support influences mobility decisions of the ones in need, and how this benefits the lives across generations, should be a central issue on the social research agenda.

## Notes

1 We could not identify mothers who – for practical or other reasons – continued to live at the same address as their ex‐partner after the relationship had ended at any point in 2008. Hence, this (probably small) group was included in the group of non‐separated women, resulting in a potential limited underestimation of separation.

2 Non‐separated mothers included those who separated after 2008, in 2009 and 2010. This group was estimated at around 2.9% (1.5% yearly). We did not consider this problematic for the comparison between non‐separated and separated, as it is a small group working conservatively for the tests of our hypotheses.

3 Moves to coresidence as a third move after separation were not included.

4 Moves of separated mothers thus took place within a time window of 2 years minimum and 3 years maximum, depending on the exact timing of separation in 2008; moves of non‐separated mothers were observed within a time window of 3 years. Because of the longer observation window, the frequency of moves among non‐separated mothers is overestimated compared with the frequency among separated mothers. However, because this results in a conservative comparison of moving probabilities and because non‐separated mothers move less often, the overestimation does not influence the results of our study.

5 Earlier studies on intergenerational support and mobility showed that the individual with support needs is usually the one who moves (Smits, 2010). This was also the case in our study: grandmothers showed no selective tendency to move near their separated daughters. Both among non‐separated and separated mothers, around 1.5% of the grandmothers moved to the mother's municipality rather than vice versa. These cases were not included in category 1 but were included in one of the other categories, in accordance with the mother's mobility behaviour.
